# Henry R. Wampole & Co.

**Published:** 1889-12

**Authors:** 


THE SOUTHERN MEDICAL COLLEGE is in prosperous operation in all of its departments, both Dental and Medical. The Hospital is also in good running order, and treating all classes of patients, Surgical, Medical and gynecological.

The annual opening of the College occurs the first week in October of each year. Parties desiring information can find it by addressing a note to Dr. W. P. Nicolson, the dean of the medical department, or Dr. Wm Crenshaw, the dean of the dental department.

